# S-wave attenuation in northeastern Sonora, Mexico, near the faults that ruptured during the earthquake of 3 May 1887 Mw 7.5

**DOI:** 10.1186/2193-1801-3-747

**Published:** 2014-12-17

**Authors:** Gina P Villalobos-Escobar, Raúl R Castro

**Affiliations:** Centro de Investigación Científica y de Educación Superior de Ensenada (CICESE), División Ciencias de la Tierra, Departamento de Sismología, Carretera Ensenada-Tijuana No. 3918, Ensenada, Baja California 22860 México

**Keywords:** Seismic attenuation, Geometrical spreading, Sonora, Mexico

## Abstract

We used a new data set of relocated earthquakes recorded by the Seismic Network of Northeastern Sonora, Mexico (RESNES) to characterize the attenuation of *S*-waves in the fault zone of the 1887 Sonora earthquake (*M*_*w*_ 7.5). We determined spectral attenuation functions for hypocentral distances (*r*) between 10 and 140 km using a nonparametric approach and found that in this fault zone the spectral amplitudes decay slower with distance at low frequencies (*f* < 4 Hz) compared to those reported in previous studies in the region using more distant recordings. The attenuation functions obtained for 23 frequencies (0.4 ≤ *f* ≤ 63.1 Hz) permit us estimating the average quality factor *Q*_*S*_ = (141 ± 1.1 )*f*^(0.74 ± 0.04)^ and a geometrical spreading term *G*(*r*) = 1/*r*^0.21^. The values of *Q* estimated for *S*-wave paths traveling along the fault system that rupture during the 1887 event, in the north–south direction, are considerably lower than the average *Q* estimated using source-station paths from multiple stations and directions. These results indicate that near the fault zone *S* waves attenuate considerably more than at regional scale, particularly at low frequencies. This may be the result of strong scattering near the faults due to the fractured upper crust and higher intrinsic attenuation due to stress concentration near the faults.

**Electronic supplementary material:**

The online version of this article (doi:10.1186/2193-1801-3-747) contains supplementary material, which is available to authorized users.

## Introduction

On May 3, 1887, a mayor seismic event (*M*_*w*_ = 7.5) took place on northeastern Sonora (Mexico), destroying the town on Bavispe and its surroundings (Aguilera, 1888). This event, as well as most northern Mexico, is located south of the Basin and Range province (Suter and Contreras, 2002), and generated the longest recorded normal fault surface rupture (101.8 km) in historic time (Suter, 2006).

The Red Sísmica del Noreste de Sonora (RESNES) was installed in 2002 to monitor the seismicity related with the Basin and Range normal faults of northeastern Sonora (Castro *et al.*, 2002; Romero *et al*., 2004). The seismic instruments of the RESNES array consist of Kinemetrics digital recorders (model K2) with internal Episensors that record the three components of ground acceleration, sampled at 200 samples per second, and an external short-period seismometer (model L4C) to record the vertical ground velocity. All stations are autonomous and have a GPS built-in timing system (Castro *et al.*, 2010).

Since the occurrence of the major event in 1887, several studies have been made, including contemporary field studies (Goodfellow, 1888; Aguilera, 1888), studies of intensity and attenuation (DuBois and Smith, 1980; Sbar and DuBois, 1984; Bakun, 2006; Castro *et al*., 2008; Castro *et al*., 2009), regional seismotectonics (Suter and Contreras, 2002), geomorphology (Bull and Pearthree, 1988; Pearthree *et al*., 1990) and microseimicity (Natali and Sbar 1982). Condori (2006) and Castro *et al*. (2008) studied the spectral amplitude decay of body waves with hypocentral distance and proposed local and regional attenuation curves to characterize the attenuation near the fault zone and at distances beyond 100 km from the center of the network.

An important element to evaluate seismic hazard is the attenuation, particularly to estimate the intensity of ground-motion at different epicentral distances. Thus, the quality factor *Q* and the geometrical spreading are key parameters for ground-motion predictions and for seismic hazard analysis. We use in this article a new and more complete data set to study in more detail the seismic attenuation near the fault zone. This new data set is composed by earthquakes relocated by Castro *et al*. (2010) near the faults that rupture during the 1887 event. We also compare results from previous studies of spectral attenuation in the Sonora region with our new results and with results from other studies within the Basin and Range province.

## Data

**Figure 1:**
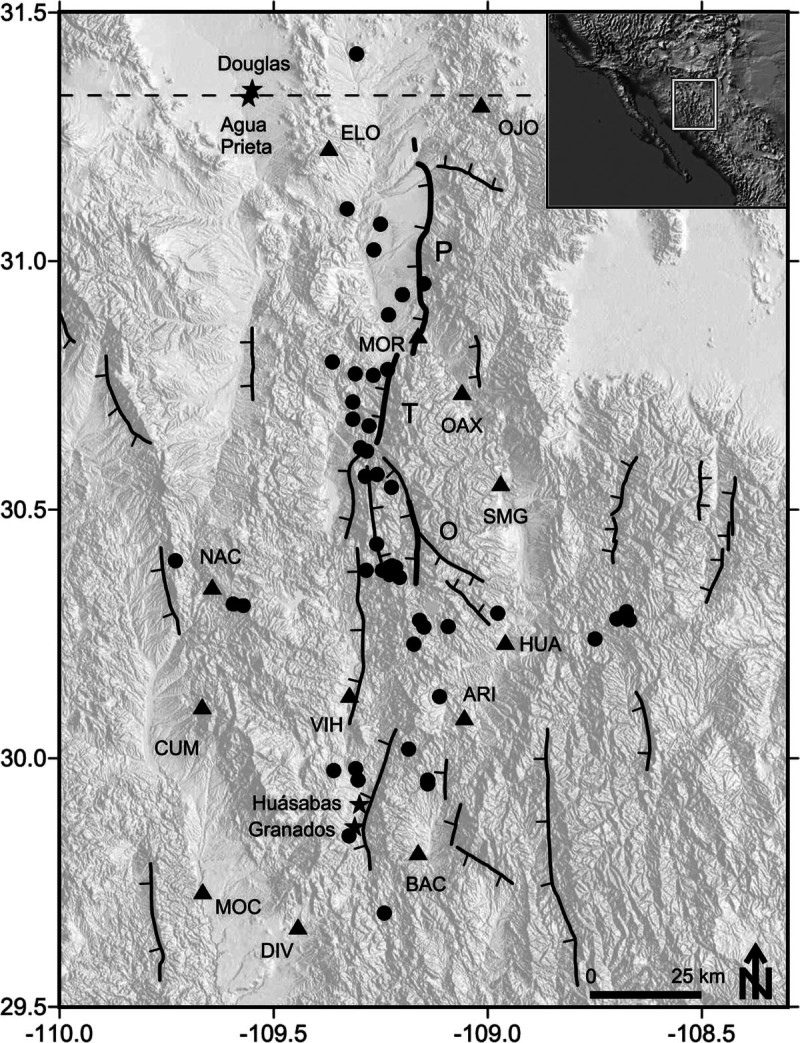
**Tectonic map showing location of stations and earthquakes used.** Black circles represent the events used for the attenuation study and triangles the recording stations. The stars represent centers of population. P (Pitáycachi), T (Teras), and O (Otates) are the faults that ruptured in the 1887 earthquake. Modified from Castro et al. (2010).

**Figure 2:**
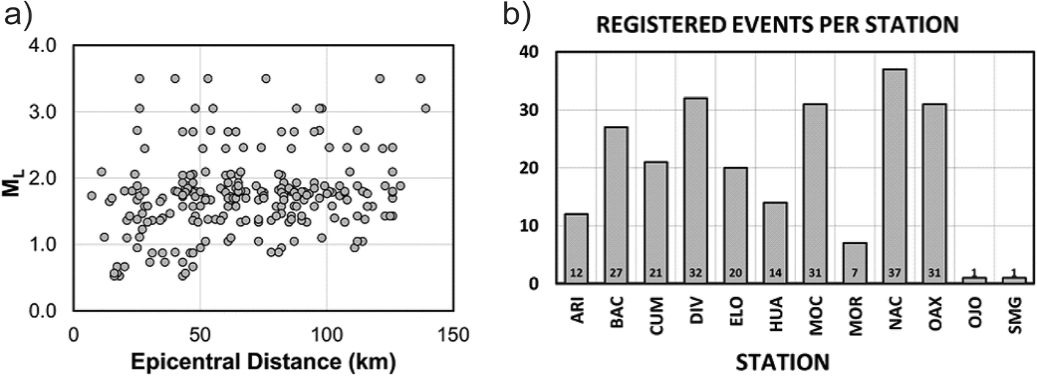
**Data set used. a)** Distribution of earthquake magnitude with epicentral distance. **b)** Histogram of ecents recorded by station.

We selected 50 events, 398 horizontal (north–south and east–west components) and 199 vertical acceleration records recorded by RESNES from 2003 to 2007 that were located near the fault zone area. Figure 1 shows the location of the earthquakes used (black circles) and the main faults of the region. The fault segments shown with thicker lines are the segments that ruptured during the 1887 events, the Pitáycachi (P), Teras (T) and Otates (O) faults. The stations of the RESNES array (triangles) are distributed around these faults. These events were relocated by Castro *et al*. (2010), have local magnitudes between 0.5 and 3.5, hypocentral distances between 10 and 140 km and focal depths of less than 40 km. Table 1 lists the source coordinates and magnitudes of the events used and Table 2 the station coordinates. Figure 2a and b show the distribution of the magnitudes with epicentral distance and the histogram of the number of records per station, respectively. Most events have magnitudes between 1 and 2 (Figure 2) and were recorded by stations NAC and OAX, located in the center of the array.

**Table 1:** **List of earthquakes used in the attenuation analysis**

Event	Date	Time	Lat(°)	Lon(°)	H (km)	Ml
**1**	19/05/2003	8:51:28	30.545	-109.2255	20.96	1.4
**2**	25/07/2003	7:53:12	31.1044	-109.3282	12.18	1.7
**3**	28/07/2003	02:39:41	30.7819	-109.2343	9.02	2.1
**4**	11/08/2003	06:59:45	30.7703	-109.2671	4.66	1.7
**5**	20/12/2003	11:34:54	30.9541	-109.1493	6.99	1.1
**6**	05/02/2004	14:11:05	31.4168	-109.3065	0.00	1.4
**7**	06/02/2004	10:01:07	29.9788	-109.308	9.46	3.1
**8**	11/03/2004	05:54:11	30.5666	-109.286	0.34	1.4
**9**	06/06/2004	10:47:06	30.8919	-109.2318	7.09	3.5
**10**	25/06/2004	12:24:02	30.6681	-109.2775	2.56	2.7
**11**	04/10/2004	09:33:25	30.3829	-109.2218	4.15	2.7
**12**	04/10/2004	09:40:12	30.4305	-109.2598	0.00	1.8
**13**	04/10/2004	09:43:18	30.369	-109.2296	0.00	1.8
**14**	04/10/2004	09:51:00	30.3835	-109.2191	0.00	1.6
**15**	04/10/2004	10:06:25	30.3848	-109.2294	0.00	2.1
**16**	04/10/2004	10:23:53	30.3864	-109.2241	0.00	1.9
**17**	04/10/2004	11:31:53	30.3781	-109.2461	21.63	1.9
**18**	04/10/2004	11:35:17	30.3798	-109.2241	0.00	1.8
**19**	04/10/2004	17:08:56	30.3836	-109.2145	4.75	1.7
**20**	26/10/2004	17:30:17	30.3072	-109.5717	7.80	1.2
**21**	28/10/2004	02:23:05	30.2779	-108.6694	3.73	2.5
**22**	28/10/2004	02:29:18	30.2948	-108.6765	2.16	1.8
**23**	05/11/2004	22:13:13	30.0185	-109.1854	2.51	2.1
**24**	14/11/2004	00:47:09	29.975	-109.3597	1.81	0.7
**25**	01/12/2004	09:52:48	29.6885	-109.2416	2.01	0.7
**26**	03/12/2004	05:32:21	30.2886	-108.6797	6.63	1.8
**27**	03/12/2004	09:10:01	30.2798	-108.6987	2.82	1.9
**28**	06/12/2004	16:14:39	29.8437	-109.3243	6.50	1.8
**29**	02/01/2005	11:37:33	30.2395	-108.7495	6.07	1.8
**30**	08/01/2005	01:22:14	30.2639	-109.1491	28.92	1.4
**31**	10/01/2005	12:43:19	31.0746	-109.2501	4.33	1.8
**32**	10/02/2005	15:34:11	30.3095	-109.5942	6.96	1.7
**33**	24/03/2005	16:49:34	30.7731	-109.3092	2.74	2.5
**34**	05/07/2005	05:27:26	29.956	-109.3033	5.82	0.9
**35**	27/09/2005	12:30:25	30.2288	-109.1722	3.01	1.4
**36**	28/10/2005	14:27:04	29.9487	-109.1408	3.01	0.5
**37**	30/10/2005	11:03:59	29.956	-109.1404	3.99	0.6
**38**	16/11/2005	04:17:40	30.2769	-109.1589	2.77	1.7
**39**	02/01/2006	02:20:03	30.6242	-109.2976	0.00	1.3
**40**	05/01/2006	22:21:31	30.2643	-109.092	14.84	1.1
**41**	06/01/2006	18:56:00	30.9326	-109.1992	16.05	1.5
**42**	16/01/2006	07:20:44	30.2914	-108.977	22.84	1.0
**43**	28/03/2006	09:19:41	30.7165	-109.3152	0.00	1.6
**44**	16/05/2006	23:47:05	30.5705	-109.2579	3.85	1.6
**45**	17/05/2006	05:34:21	30.1241	-109.1125	2.02	1.7
**46**	29/06/2006	09:46:47	30.6819	-109.3154	0.68	1.1
**47**	05/08/2006	12:33:26	30.6181	-109.2829	2.00	1.8
**48**	13/10/2006	04:23:38	31.0228	-109.2666	5.86	1.4
**49**	19/10/2006	18:00.2	30.3781	-109.2839	17.22	0.9
**50**	21/02/2007	13:45:28	30.7968	-109.3635	17.33	1.4

**Table 2:** **Station coordinates**

Station	Lat(°N)	Long(°W)	Elevation (m)
**ARI**	30°04.93	109°03.30	1546
**BAC**	29°48.67	109°09.78	70
**CUM**	30°06.27	109°40.02	1139
**DIV**	29°39.71	109°26.78	745
**ELO**	31°13.65	109°22.25	121
**HUA**	30°14.06	108°57.52	945
**MOC**	30°44.19	109°39.92	62
**MOR**	30°51.07	109°09.76	99
**NAC**	30°20.74	109°38.60	126
**OAX**	30°44.19	109°03.63	90
**OJO**	31°18.90	109°00.90	145
**SMG**	30°33.19	108°58.19	94

**Figure 3:**
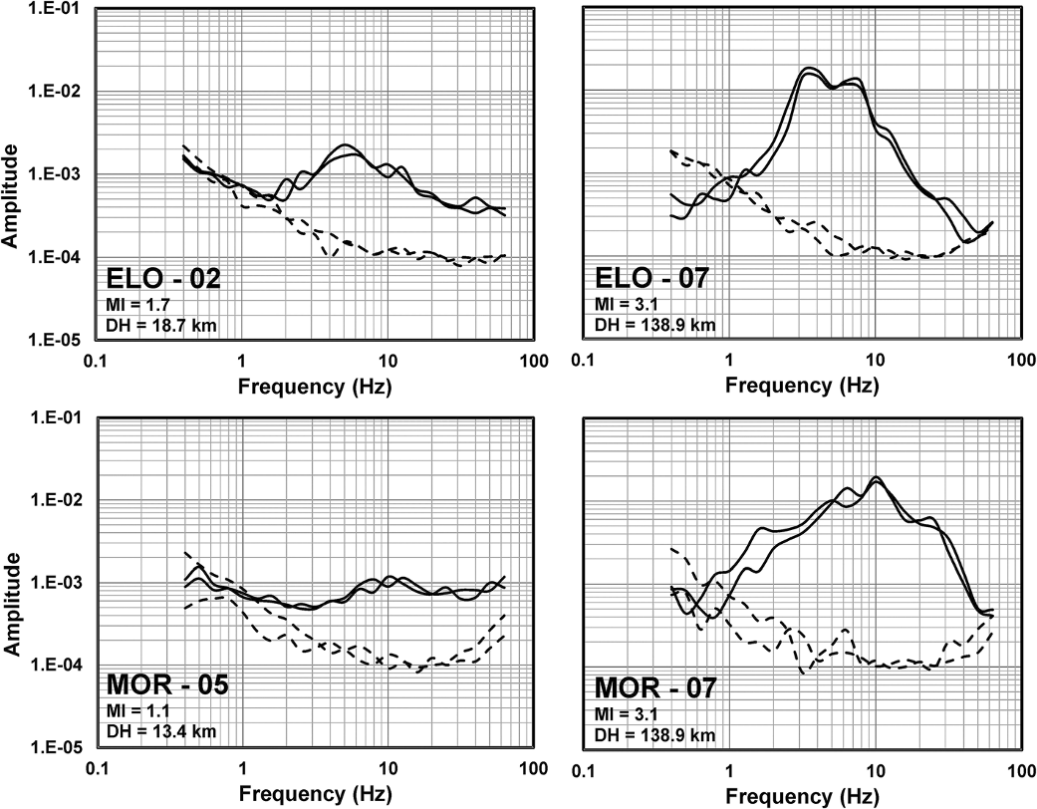
**Examples of signal and noise spectra from stations ELO (located on bedrock) and MOR (located on conglomerate).** Left panels are spectra from local events (r < 20 km) and right panels spectra from regional events (r = 138.9 km). Continuous lines represent the 80% energy *S*-wave spectra (both horizontal components) and discontinuous lines are the noise spectra of a 6-seconds time window choose previous to the first *P*-wave arrival.

**Figure 4:**
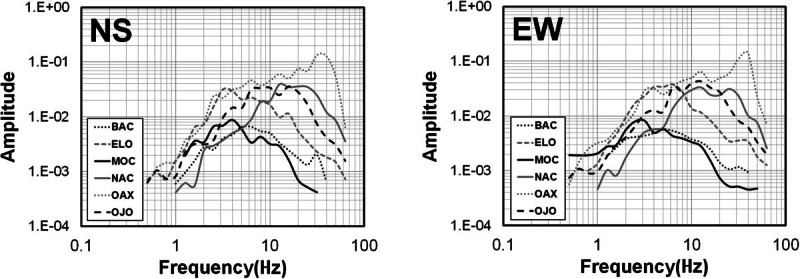
**Acceleration spectra of horizontal components of event 9 (** ***M***_***L***_ **= 3.5).**

**Figure 5:**
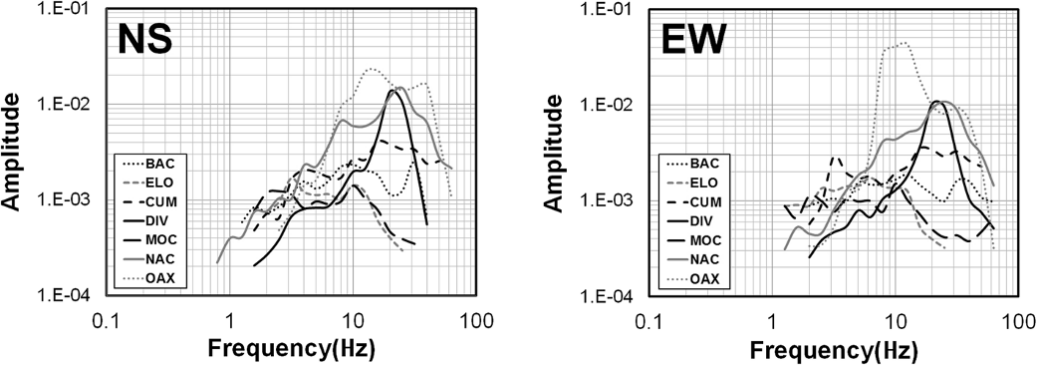
**Acceleration spectra of horizontal components of event 16 (** ***M***_***L***_ **= 1.9).**

The records were baseline corrected, subtracting the mean, to remove long-period biases. We chose time windows starting before the first *S*-wave arrival and containing 80% of the *S*-wave energy to calculate the Fourier acceleration spectra of the north–south (NS) and east–west (EW) components. We verify visually that the windows contain the strong-ground motions including the peak acceleration. The first and last 5% of the window are cosine tapered and the spectral amplitudes smoothed averaging within a variable frequency band of ±25% of 23 predefined central frequencies between 0.4 and 63.1 Hz. The spectral amplitudes were smoothed using a variable frequency band of +/- 25% over the 23 predefined central frequencies. For each spectral record, we selected the signal frequency band above noise level by visual inspection of the spectrum. Figure 3 shows examples of signal and noise spectra from stations ELO and MOR located on bedrock and conglomerates, respectively. This figure compares noise and signal spectral levels recorded at both horizontal components from events recorded at epicentral distances less than 20 km (left frames) and at 138.9 km (right frames). The signal spectra (solid lines) correspond to the 80% of the energy of the *S*-waves and the noise spectra (dashed lines) to 6-seconds time windows starting before the first *P*-wave arrivals. Examples of the acceleration spectra calculated for two events with magnitudes *M*_*L*_ 3.5 and 1.9 are also shown in Figures 4 and 5, respectively. The signal to noise ratio for these stations is above one in the frequency band 1.0 – 63.1 Hz for local events but increases for events with lager magnitude (M > 1.7). For regional events the signal to noise ratio of stations ELO and MOR is above one in the band 1.0 – 40 Hz.

## Method

### Attenuation functions using a nonparametric method

We explore the dependence of the spectral amplitudes with hypocentral distance by considering that for a fixed frequency, the spectral amplitudes can be represented by empirical determined functions obtained following a model that separates source size from attenuation effects (e.g. Castro *et al*., 1990; Anderson and Lei, 1994; Castro *et al*., 1996; Castro *et al.*, 2008). This technique was first proposed by Brillinger and Preisler (1984) for the analysis of attenuation relations of peak accelerations. The spectral amplitudes can be modeled with the following equation:

1 $$U_{i}\left(f,r\right)=S_{i}\left(f\right)A\left(f,r\right)$$

Where *U*_*i*_(*f*, *r*) is a datum from event *i* recorded at hypocentral distance *r* at a frequency *f, S*_*i*_(*f*) is a scalar that depends on the size of the *i* th event at a frequency *f. A*(*f*, *r*) is the empirically determined attenuation function that describes the distance decay trend. We assume that *A*(*f*, *r*) implicity contains the effect of both the geometrical spreading and the quality factor *Q*, but we do not limit their behavior to a predetermined parametrical function, instead, we constrain *A*(*f*, *r*) to decay smoothly with distance. The basis of the smooth decaying restriction lies on the principle that the inelastic properties in the crust tend to decrease the amplitudes gradually with distance and that undulations may be related to other factors such as site and wave propagation effects that would reflect on the residuals when solving equation (1) (Castro *et al*., 1990; Castro *et al*., 2008).

The main assumptions of the model described by equation (1) are that *A*(*f*, 0) = 1.0, because at *r* = 0 there is not attenuation and the spectral amplitudes are fully governed by the source term *S*_*i*_(*f*). The model assumes that the shape of the attenuation function is the same for all the sources, for a given frequency, regardless of the size of the event (Castro *et al*., 1990; Castro *et al*., 2008). Thus, the source factor *S*_*i*_(*f*) shifts upwards or downwards the attenuation function depending on the magnitude of the event without modifying its shape. One major advantage of this last assumption is that the observed amplitudes of events recorded at different distances at a given frequency complement each other and permits to define the attenuation function at a wider distance range.

To solve equation (1), we took logarithms at both sides of the equation and formed a set of linear equations for a given frequency of the form:

2 $$u_{ij}=s_{i}+a_{j}$$

Where *u*_*ij*_ = *logU*_*i*_(*f*, *r*) is a datum from earthquake *i* at distance *j*, *s*_*i*_ = *logS*_*i*_(*f*), and *a*_*j*_ = *logA*(*f*, *r*) is the value of the attenuation function at distance *j*. We discretized the distance range of the observations using a constant interval. Equation (2) represents a system of equations that is solved by a constrained least-squares inversion. Equation (2) can be expressed on matrix form as:

3 $$\left(\begin{array}{ccccc} 1 & 0 & 0 & . & .\\ 0 & 1 & 0 & . & .\\ . & . & . & . & .\\ . & . & . & . & .\\ w_{1} & 0 & 0 & . & .\\ -w_{2}/2 & w_{2} & -w_{2}/2 & 0 & .\\ 0 & -w_{2}/2 & w_{2} & . & .\\ . & . & . & . & . \end{array}\right)\cdot \left(\begin{array}{c} a_{1}\\ .\\ .\\ a_{j}\\ s_{1}\\ .\\ .\\ s_{i} \end{array}\right)=\left(\begin{array}{c} u_{11}\\ .\\ .\\ u_{ij}\\ 0\\ 0\\ .\\ \text{.} \end{array}\right)$$

Where *w*_1_ and *w*_2_ are weighting factors provided as input to constrain *a*_1_ *= 0* at *r = 0*, and to weight the second derivate, for smoothing purposes, respectively. The weighting factor *w*_1_ forces the attenuation function to cross the origin. We tested diverse values of the weighting factors and discretized the hypocentral distances at different intervals in order to achieve the expected monotonic and smooth decay of the attenuation function without losing the characteristic trend of the data.

**Figure 6:**
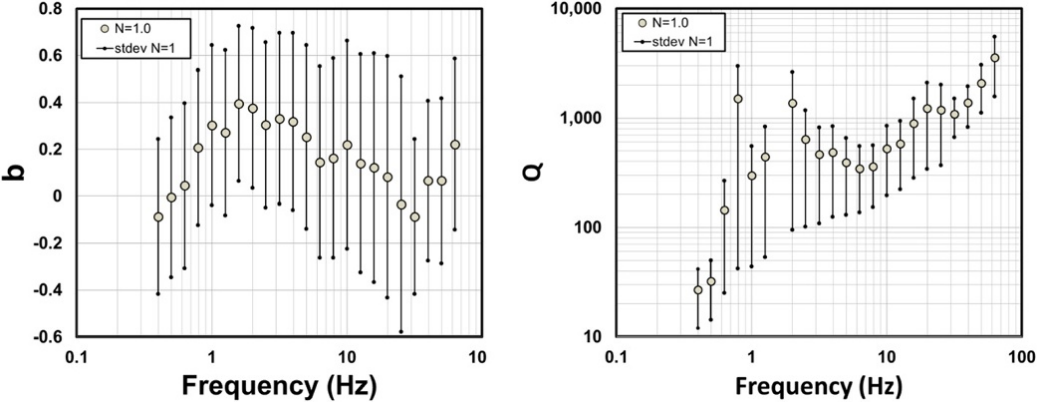
**Examples of nonparametric attenuation functions obtained for 10 different frequencies.** Black circles are observed horizontal *S*-wave spectral amplitudes for all magnitudes, open circles are observed vertical *S*-wave spectral amplitudes for all magnitudes. Black continuous line corresponds to the attenuation function found for the horizontal spectral amplitudes (cm/s^2^) and dashed line corresponds to the attenuation function found for the vertical component of the acceleration spectral amplitudes (cm/s^2^).

**Figure 7:**
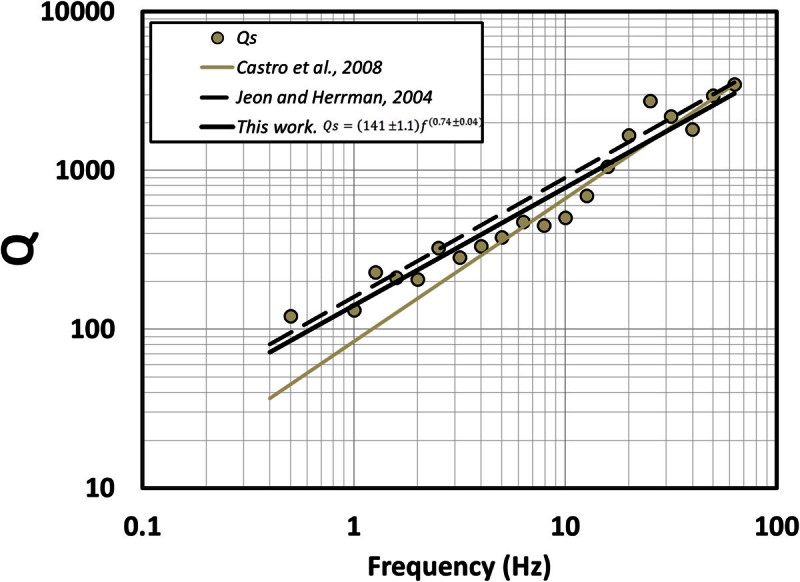
**Attenuation functions obtained for event 9 (** ***M***_***L***_**=3.5) for 10 of the 23 frequencies studied.** The dots are the observed horizontal spectral amplitudes of event 9, continuous line represents the un-scaled attenuation function and the dashed line represents the attenuation function scaled by its respective source-size factor.

The resulting model parameters *a*_*j*_ define the shape of *A(f,r)* for each frequency considered*.* A more detailed description of this method can be found in Castro *et al.*, (1990 and 1996). Figure 6 shows examples of attenuation functions found for 10 of the 23 frequencies (0.40-63.1 Hz) analyzed and the respective spectral amplitude data used to calculate them. The amplitudes in Figure 6 were scaled according with the value of *s*_*i*_ obtained from the inversion. Figure 7 shows spectral amplitudes from event 9 (*M*_*L*_ 3.5) and the corresponding attenuation function (dashed line) scaled with the value of *s*_*9*_ obtained. This figure illustrates how good the attenuation function obtained for event 9 fits the observed spectral amplitudes.

### The quality factor Q using a homogeneous attenuation model

The attenuation functions *A(f,r)* can be used to analyze various sources of *S*-wave attenuation and to estimate the quality factor *Q*. With this purpose, we model these empirically determined functions as the product of two different attenuation mechanisms (Castro *et al*., 1999):

4 $$A\left(f,r\right)=\frac{N}{r^{b}}\text{exp}-\left(\frac{\pi f\left(r-N\right)}{\upsilon Q}\right)$$

Where *1/r*^*b*^ represents the effect of geometric spreading, *N* is a normalization distance and the exponential function accounts for the amplitude decrease due to total *Q* (intrinsic plus scattering). The normalization distance *N* is a reference distance that is chosen as close to the source as the data permits. Note that (*r* – *N*) in equation (4) is the *S*-wave path distance length where *Q* takes effect to normalize the spectral amplitudes at the reference distance. The parameter υ is the average *S*-wave velocity (3.4 km/sec), based on the crustal structure reported by Harder and Keller (2000) for this region. This model has a crustal thickness of 35 km and consists of three layers: the uppermost layer is one km thick and has a *S*-wave velocity of 2.86 km/s; the second layer has a thickness of 21 km and a velocity of 3.3 km/s; and the third layer, that represents the lower crust, has a thickness of 13 km and a velocity of 3.88 km/s. Different values of *N*, between 1 and 20 km, were tested to evaluate the influence of this parameter in the geometric spreading behavior.

The parameters *b* and *Q* can be estimated for each frequency by linearizing equation (4), taking logarithms at both sides. Thus, for a given frequency *f* we can rewrite equation (4) as a set of linear equations of the form:

5 $$d_{i}=c_{i}b+m_{i}1/Q$$

Where *d*_*i*_ *=* log *A(f,r*_*i*_*) –* log*N* is the normalized amplitude at distance *r*_*i*_, *m*_*i*_ *= -πf(r*_*i*_*-N)loge/υ, υ =* 3.4 km/s is the average *S*-wave velocity and *c*_*i*_ *= -log(r*_*i*_*). 1/Q* and *b* are estimated by solving equation (5) with a least-squares inversion scheme.

## Results and discussion

### Attenuation functions

The spectral amplitudes modeled with equation (1) describe the *S*-wave attenuation in northeastern Sonora, in the region close to the rupture zone of the 1887 (*M*_*W*_ = 7.5) earthquake. We determined 23 spectral attenuation functions that represent the decay of the *S*-wave energy for discrete frequencies defined between 0.4 and 63.1 Hz. Figure 6 shows a sample of these attenuation functions and the observed spectral amplitudes for both horizontal (black circles) and vertical (open circles) components. We observe that spectral amplitudes at low frequencies (up to 5.0 Hz) tend to attenuate less with hypocentral distance than amplitudes at higher frequencies. Figure 7 compares the observed spectral amplitudes (circles) from a *M*_*L*_ = 3.5 earthquake (event 9 in Table 1) with the nonparametric attenuation functions (dashed lines) scaled with the corresponding values of *S*_*i*_*(f)* (equation (1)) resulting from solving equation (2). We also plotted in Figure 7*A(f, r)* for *S*_*i*_*(f) = 1*(solid lines) as a reference to show that for a given frequency the shape of the attenuation function is the same regardless of the event size. In other words, the rate of decay of the spectral amplitudes with distance is assumed to be the same for all the events.

To evaluate the effect of site conditions of the recoding stations, we calculated spectral attenuation functions separately for both vertical and horizontal ground-motion components using the whole distance range (Figure 6). Under the assumption that the vertical component of acceleration is less susceptible to site-amplification effects, a comparison of the attenuation functions obtained using the vertical component provides a reference to evaluate the effect of local site response on the attenuation functions determined with the horizontal components. We found no substantial differences between the observed spectral data or between the calculated attenuation functions (Figure 6).

### Estimates of Q

**Figure 8:**
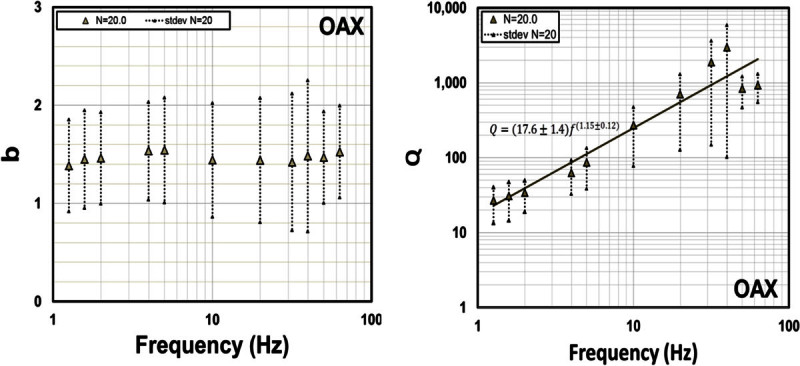
**Estimates of** ***b*** **(left frame) and** ***Q*** **(right frame) for** ***N = 1.0*** **(see equation (** 4**)) for the whole hypocentral distance range (10-140 km).**

The attenuation functions can be modeled with three parameters: *N*, *b* and *Q* (equation 4)_*.*_*N* is a reference distance chosen as the closest source-station distance, *b* define the rate of decay due to geometrical spreading, and *Q* accounts for the attenuation due to anelasticity, friction losses, multipathing and scattering effects. Figure 8 shows estimates of *b* and *Q*, calculated for *N =* 1, obtained using the attenuation functions for the hypocentral distance range between 10 and 140 km, and the horizontal acceleration spectra. The values of *Q* (Figure 8), obtained making *N* = 1, increase with frequency from 27 at 0.4 Hz to 3547 at 63.1 Hz, while the values of *b* show a weak dependence with frequency. We also calculated *Q* using values of *N* of 10 and 20 finding negative values of *Q* for most frequencies. Thus, we consider that *N* = 1 is an adequate reference distance, since provides positive values of *Q* and a good fit to the observed spectral amplitude decay.

**Figure 9:**
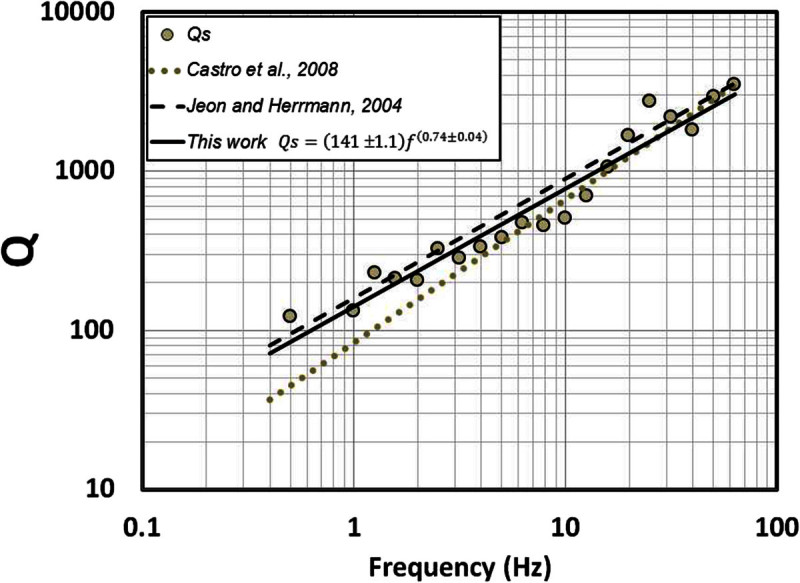
**Estimations of** ***Q*** **for** ***S*** **waves in the region close to the fault zone of the 1887 earthquake.** Black continuous line is the mean-square fit of the *Q* values (circles) using *b* = 0.21 obtained in this work. Grey dotted line corresponds to the regional estimation proposed by Castro et al. (2008). Discontinuous black line corresponds to the values of *Q* obtained by Jeon and Herrmann (2004) for the Basin and Range province in Utah (USA).

Although the idealized far-field body wave geometrical spreading in a homogeneous whole space is given by *G*(*r*) = *r*^- 1^, some studies (Ou and Herrmann 1990; Burger *et al*., 1987; Chapman and Godbee, 2012 Morozov, 2010) indicate that the apparent geometrical spreading in velocity structures other than a homogeneous whole space will generally involve more complex amplitude decay than *1/r*. The free parameter *b* in equation (4) can take different values at different frequencies and accounts for a geometrical spreading that may depart from the value expected for a homogeneous medium. If we adopt a constant geometric spreading model with *b = 0.21*, that corresponds to the average value of *b* in the frequency band of 0.5 – 63.1 Hz, and recalculate *Q* (Figure 9 and Table 3), the frequency dependence of *Q* can be approximated with the relation

**Table 3:** **Values of** ***Q*** **(Figure**9**) estimated at different frequencies using** ***N*** **= 1 and** ***b*** **= 0.21 in equation (** 4**) with the RMS values reported by the inversion process**

Frecuency (Hz)	***Q***	RMS values
0.50	121.3	0.358
1.00	132.6	0.385
1.26	229.0	0.413
1.58	212.6	0.399
2.00	206.5	0.438
2.51	325.3	0.430
3.16	282.8	0.418
3.98	334.3	0.433
5.01	381.7	0.426
6.31	475.6	0.401
7.94	452.3	0.376
10.0	504.5	0.381
12.59	695.7	0.377
15.85	1054.0	0.367
19.95	1672.0	0.373
25.12	2747.0	0.403
31.62	2201.0	0.412
39.81	1819.0	0.381
50.12	2955.0	0.364
63.10	3498.0	0.359

6 $$Q_{S}=\left(141\pm \;1.1\;\right)f^{\left(0.74\;\pm \;0.04\right)},\;\left(0.5\leq f\leq \;63.1\;\mathrm{Hz}\right)$$

Castro *et al.* (2008) calculated attenuation functions of body waves for a wider region using a smaller data set and found that

7 $$Q_{S}=84f^{0.9},\;\left(0.5\leq f\leq \;63.1\;\mathrm{Hz}\right)$$

Although the model they used to describe the sources of attenuation involve a term that accounts for the near-surface attenuation, their *Q*-frequency relation (equation (7)) follows the general trend of *Q* obtained in this study (circles in Figure 9). However, between 0.63 Hz and 4.0 Hz equation (7) underestimates the values of *Q* here determined (Figure 9). The data sets used in previous studies (Castro *et al*., 2008, 2009) and that used in this paper have important differences. First, we used a greater number of events located in the epicentral area of the 3 May 1887 *M*_*W*_ 7.5 earthquake; second, we used hypocenters relocated by Castro *et al*. (2010) and the precise locations guarantee a better control on the source-station distances; third, the volume sampled by the source-station paths in this paper follows the strike of the faults closer than previous studies.

Jeon and Herrmann (2004) estimated a *Q*-frequency relation similar to equation (6) with a geometric spreading coefficient *b* defined by sections depending on the hypocentral distance (*b* = 1.2 for 0 < r ≤ 50 km; *b =* 0.55 for 50 ≤ r ≤ 60 km; *b =* 0.2 for 60 ≤ r ≤ 90 km; *b =* 0.1 for 90 ≤ r ≤ 140 km and *b =* 0.5 for 140 < r ≤ 500 km) in the Basin and Range Province in Utah (USA). This function overestimates slightly the values of *Q* that we estimated between 3 and 16 Hz (Figure 9), but in general their relation:

8 $$Q_{S}\left(f\right)\;=\;160\;f^{0.75}$$

follows a similar trend of our *Q* estimates. The parameter *b* depends on the hypocentral distance, and for distances greater than 50 km has values below the theoretical value of one.

### Attenuation in the north–south direction

To evaluate the attenuation of *S* waves in the north–south direction, along the strike of the faults that ruptured during the 1887, we calculated attenuation functions using data from the same site to avoid the possible influence of site amplification effects. We selected station OAX (Figure 1) for having the largest amount of records and for being located inside the fault zone. We formed a dataset with horizontal spectral records of OAX from 33 events with magnitudes between 1.0 and 3.5 that occurred in the 20–90 km distance range from the station. We determined attenuation functions for the same frequencies and procedure (equation 1) as before, and then we used them to estimate *b* and *Q* with equation (4).

**Figure 10:**
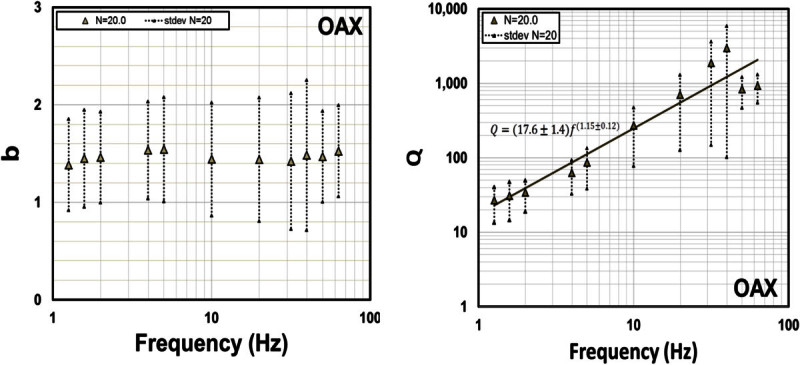
**Estimates of** ***b*** **(left frame) and** ***Q*** **(right frame) for** ***N = 20*** **obtained with records from station OAX for hypocentral distance in the range of 20–90 km** ***.***

Figure 10 displays *b* and *Q* obtained with the attenuation functions determined for station OAX, using values of *N =* 20 (the closest source-station distance in the data set). We found that *Q* increases with frequency following the function

9 $$Q=\;17.6\pm 1.4f^{1.15\pm 0.12}$$

while *b* is weakly dependent of frequency, having a value close to 1.4 for the whole frequency band analyzed (1.3–63.1 Hz).

Some studies of body wave attenuation shows that the coefficient *b* associated with the geometrical spreading is more complex than *b* = 1 and they attribute this to complex velocity structures (Ibáñez *et al.,*^1993^; Olafsson *et al*., 1998; Castro *et al*., 1999; Akıncı *et al*., 2006; Padhy, 2009). Ou and Herrmann (1990) found that *G(r)* may depend upon source depth in layered structures, while Burger *et al.,* (1987) remarked the importance of post-critical reflections from mid-lower crustal velocity discontinuities and the Moho in controlling amplitudes in certain distance ranges. Chapman and Godbee (2012) found values of the geometrical spreading in eastern North-America up to *r*^*-4*^ for the vertical components and up to *r*^*-1.5*^ for the horizontal components, which exceeds notably the theoretical standards for body waves. In our case, we are finding similar values of the parameter *b* (for *N* = 20), for the OAX data set, to those reported by Chapman and Godbee (2012) for the horizontal components (Figure 10). Malagnini *et al.,* (2002) introduced a slightly frequency-dependent geometrical spreading to model ground motion in northeastern Italy, a region where the attenuation parameters vary considerably. Akıncı *et al.* (2006) proposed geometric spreading functions that are frequency dependent on a stepped way for frequencies below and above 1.0 Hz and for a different range of distances in the Marmara (Turkey) region. The results obtained in this paper (Figures 8 and 10) show that *b* is weakly dependent of frequency and that there are certainly other factors, beyond simple theoretical models, to fully account for all the processes affecting the attenuation of *S*-waves in the Sonora region.

**Figure 11:**
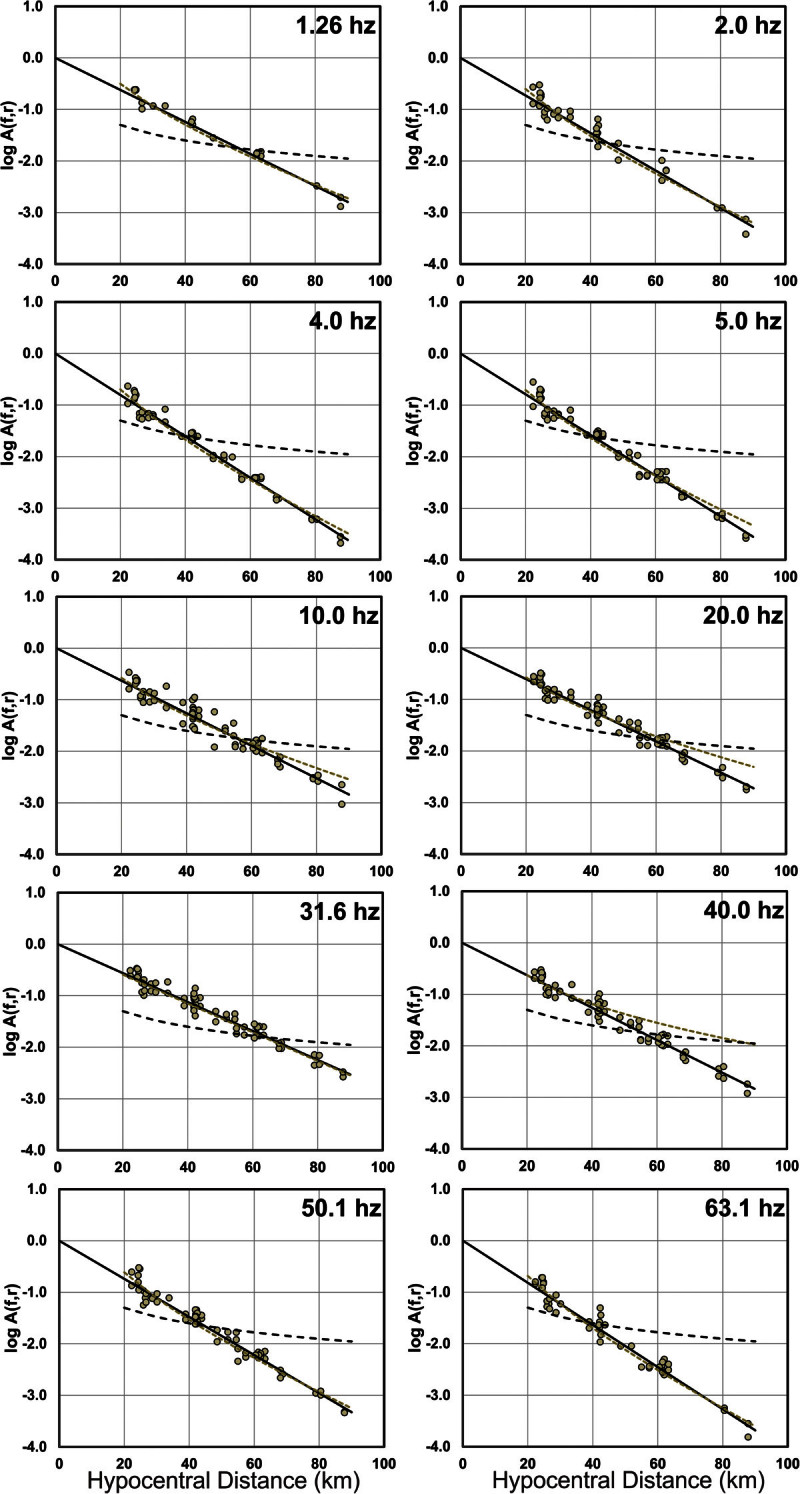
**Comparison of the nonparametric attenuation functions obtained with station OAX (continuous lines) with expected attenuation calculated using equation (** 4**) with geometric spreading** ***G*** **(** ***r*** **)** **=** ***r***^**-** ***b***^**(dotted lines) and theoretical geometric spreading** **G(r)** **=** **r**^**- 1**^**(dashed lines).** The circles are the observed horizontal spectral amplitudes used to obtain the attenuation functions.

To verify the consistency of our results, we compare in Figure 11 the nonparametric attenuation functions (continuous lines), calculated with equation (2) using the observed spectral amplitudes (circles) from station OAX, with the expected amplitude decay (grey dashed lines) calculated using equation (4) with the estimated values of *Q* and *b*, using *N* = 20 (Figure 10) for that site. Figure 11 shows also, for comparison, the geometrical spreading function *G*(*r*) = *r*^- 1^ (dashed lines). The curves calculated with equation (4), with the geometrical spreading model *G*(*r*) = *r*^- *b*^, follow the nonparametric functions very closely at all frequencies, while the function *G*(*r*) = *r*^- 1^ (dashed lines) underestimate the observed amplitudes at short distances (*r* < 40 km) and overestimates the observations at larger distances (*r* > 40 km).

## Summary and conclusions

We used precise hypocenter locations from local events to find nonparametric spectral attenuation functions for the region that ruptured with the *M*_*w*_ *=* 7.5 1887 earthquake of Sonora. The attenuation functions determined decay more slowly with hypocentral distance at low frequencies (*f* < 4 Hz) than results reported previously in the area using more distant recordings. Consequently the *Q*-frequency relation (eq. (7)) obtained by Castro *et al*. (2008) tends to underestimate *Q* between 0.63 and 4.0 Hz. The values of *Q* estimated for the *S*-waves (Figure 9) show a clear dependence of *Q* with the frequency, and agree with the models proposed by Castro *et al*. (2008) for the region, and with the values of *Q* calculated by Jeon and Herrmann (2004) for the Basin and Range Province in the state of Utah (USA).

The values of *Q* estimated with station OAX (eq. (9) and Figure 10) for *S*-wave paths traveling along the strike of the fault system located near the rupture of the 1887 event, in the north–south direction, are considerably lower than the average *Q* estimated using source-station paths from multiple stations and directions (eq. (6) and Figure 9). These results indicate that near the fault zone *S* waves attenuate considerably more than at regional scale, particularly at low frequencies. For instance, at 0.5 Hz *Q* in the north–south direction, along the strike of the faults (eq. (9)), is 10.5 times smaller than the average *Q* (eq. (6)). This may be the result of strong scattering near the faults due to the fractured upper crust and higher intrinsic attenuation due to stress concentration near the faults.

The geometric spreading models found have weak frequency dependence (Figures 8 and 10) and can be approximated as *G*(*r*) = 1/*r*^0.21^ for the average *Q*. This spreading function predicts slower amplitude decay with hypocentral distance than the *r*^- 1^ theoretical model of body waves.

## Authors’ original submitted files for images

Below are the links to the authors’ original submitted files for images. Authors’ original file for figure 1 Authors’ original file for figure 2 Authors’ original file for figure 3 Authors’ original file for figure 4 Authors’ original file for figure 5 Authors’ original file for figure 6 Authors’ original file for figure 7 Authors’ original file for figure 8 Authors’ original file for figure 9 Authors’ original file for figure 10 Authors’ original file for figure 11
