# Erratum to: S-wave attenuation in northeastern Sonora, Mexico, near the faults that ruptured during the earthquake of 3 May 1887 Mw 7.5

**DOI:** 10.1186/s40064-015-0877-y

**Published:** 2015-03-07

**Authors:** Gina P Villalobos-Escobar, Raúl R Castro

**Affiliations:** Centro de Investigación Científica y de Educación Superior de Ensenada (CICESE), División Ciencias de la Tierra, Departamento de Sismología, Carretera Ensenada-Tijuana No. 3918, Ensenada, Baja California 22860 México; Present address: Centro de Geociencias, Universidad Nacional Autónoma de México (UNAM), Blvd. Juriquilla No. 3001, Querétaro, 76230 México

## Erratum

In the original version of this article (Villalobos-Escobar and Castro 2014), Figures 6, 7, and 8 were marked up incorrectly. Figure 6 should show examples of nonparametric attenuation functions, instead the incorrect Figure 6 shows values of the exponent *b* of the geometrical spreading function (equation 4) and estimates of the quality factor *Q*. Figure 7 should show attenuation functions scaled according to event 9 (Table 1) which is an M = 3.5 earthquake. Instead, the incorrect Figure 7 printed shows estimates of *Q* obtained by other authors and those obtained in this study. Figure 8 is also incorrect; this figure should show the values of *b* and *Q* displayed in the printed Figure 6.

In this erratum, the corrected Figures 6, 7, and 8 are shown as Figure 1, Figure 2, and Figure 3, respectively.

**Figure 1:**
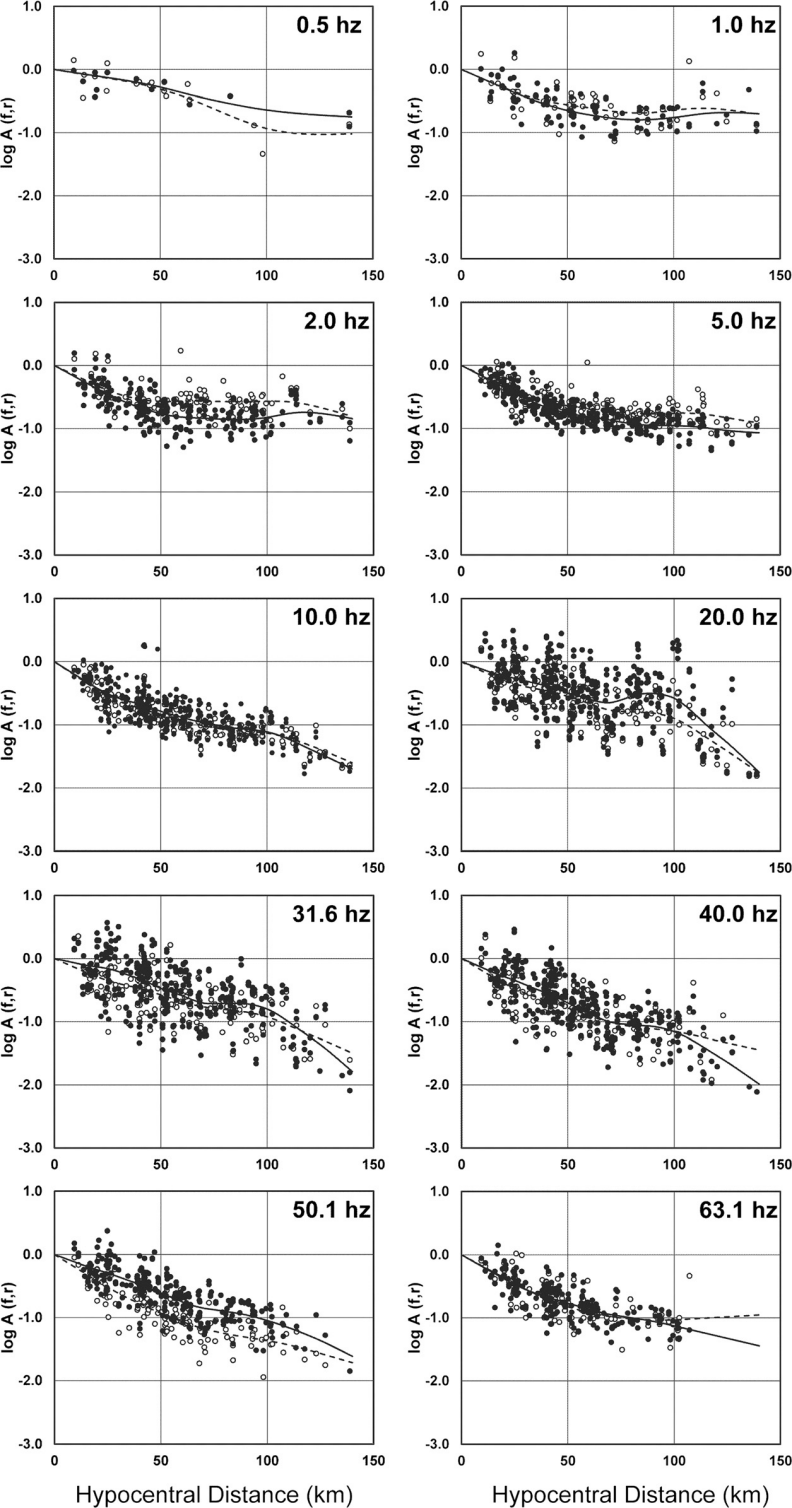
**Examples of nonparametric attenuation functions obtained for 10 different frequencies.** Black circles are observed horizontal *S*-wave spectral amplitudes for all magnitudes, open circles are observed vertical *S*-wave spectral amplitudes for all magnitudes. Black continuous line corresponds to the attenuation function found for the horizontal spectral amplitudes (cm/s^2^) and dashed line corresponds to the attenuation function found for the vertical component of the acceleration spectral amplitudes (cm/s^2^). This is the corrected Figure 6 in Villalobos-Escobar and Castro (2014).

**Figure 2:**
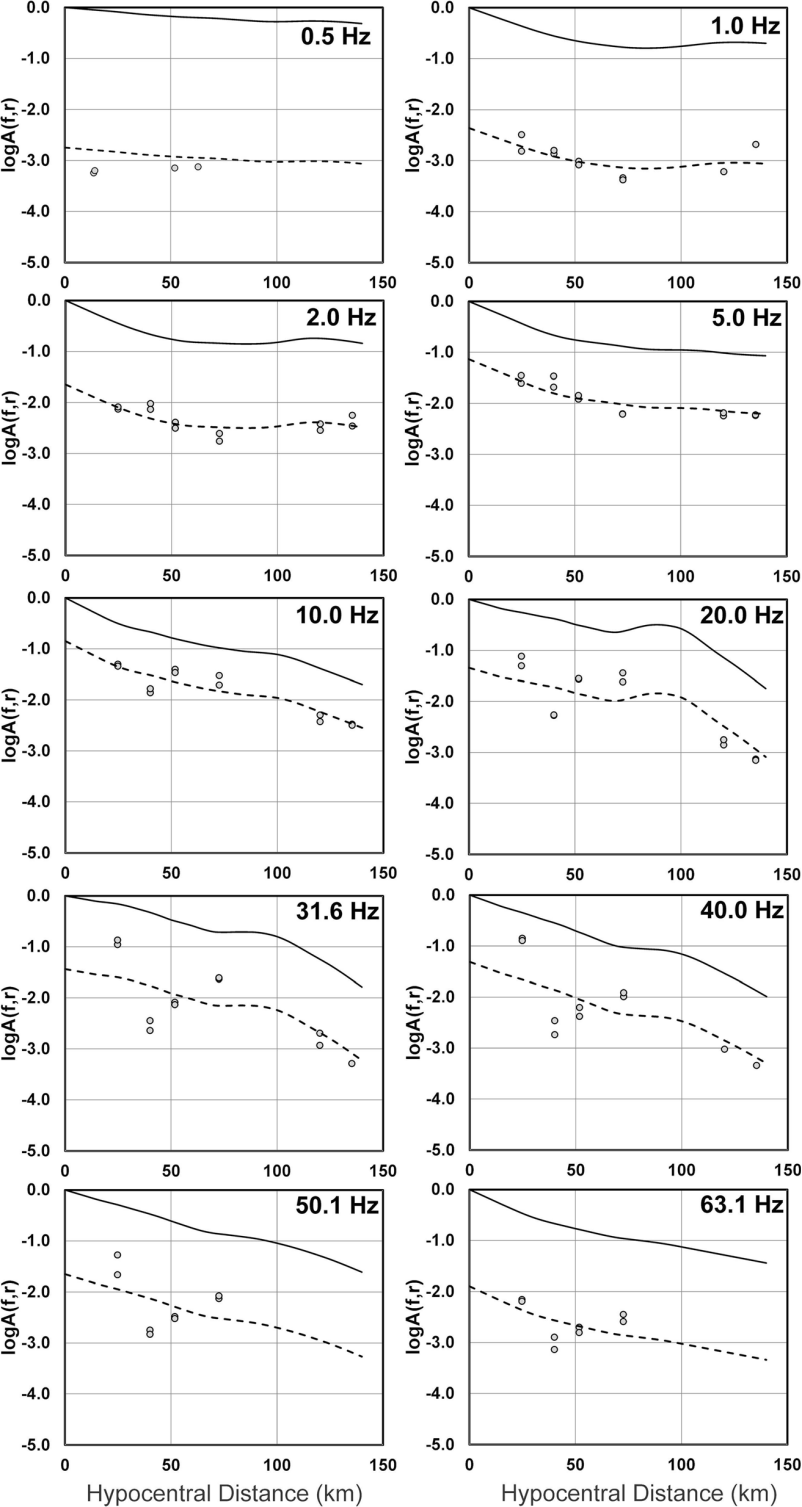
**Attenuation functions obtained for event 9 (** ***M***_***L***_ **=3.5) for 10 of the 23 frequencies studied.** The dots are the observed horizontal spectral amplitudes of event 9, continuous line represents the un-scaled attenuation function and the dashed line represents the attenuation function scaled by its respective source-size factor. This is the corrected Figure 7 in Villalobos-Escobar and Castro (2014).

**Figure 3:**
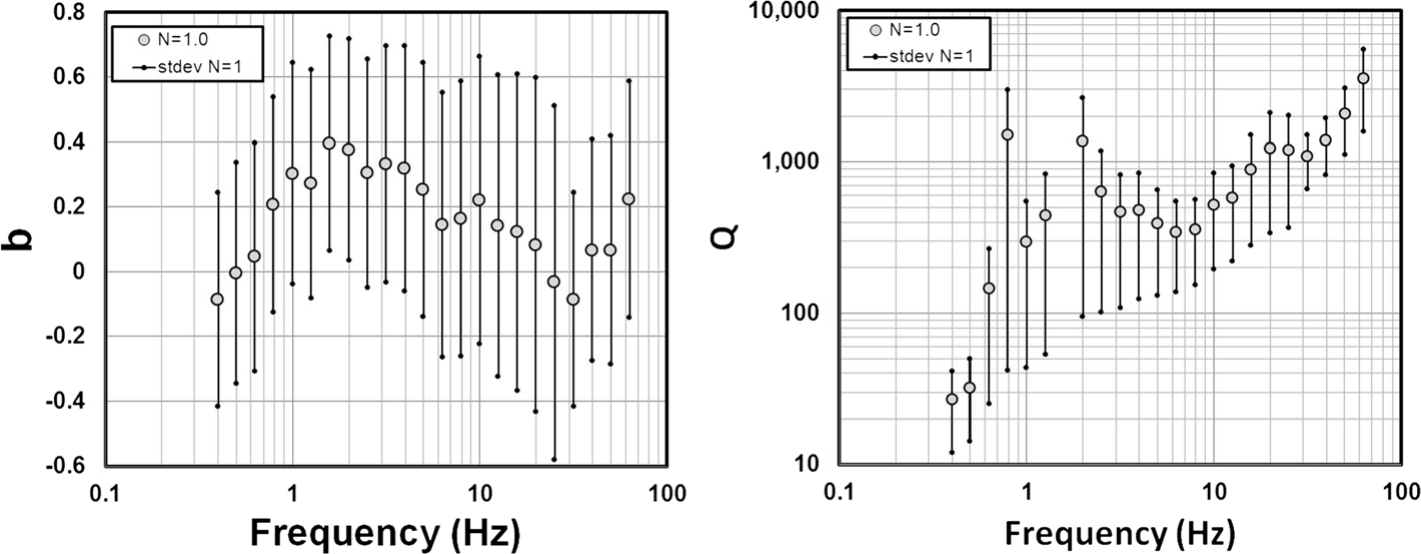
**Estimates of** ***b*** **(left frame) and** ***Q*** **(right frame) for** ***N = 1.0*** **(see equation (4)) for the whole hypocentral distance range (10-140km).** This is the corrected Figure 8 in Villalobos-Escobar and Castro (2014).
