# Supplementary figures and images for: S-wave attenuation in northeastern Sonora, Mexico, near the faults that ruptured during the earthquake of 3 May 1887 Mw 7.5
Source: Springerplus. 2014 Dec 17;3(1):747. doi: 10.1186/2193-1801-3-747 (PMC4320219; doi:10.1186/2193-1801-3-747)

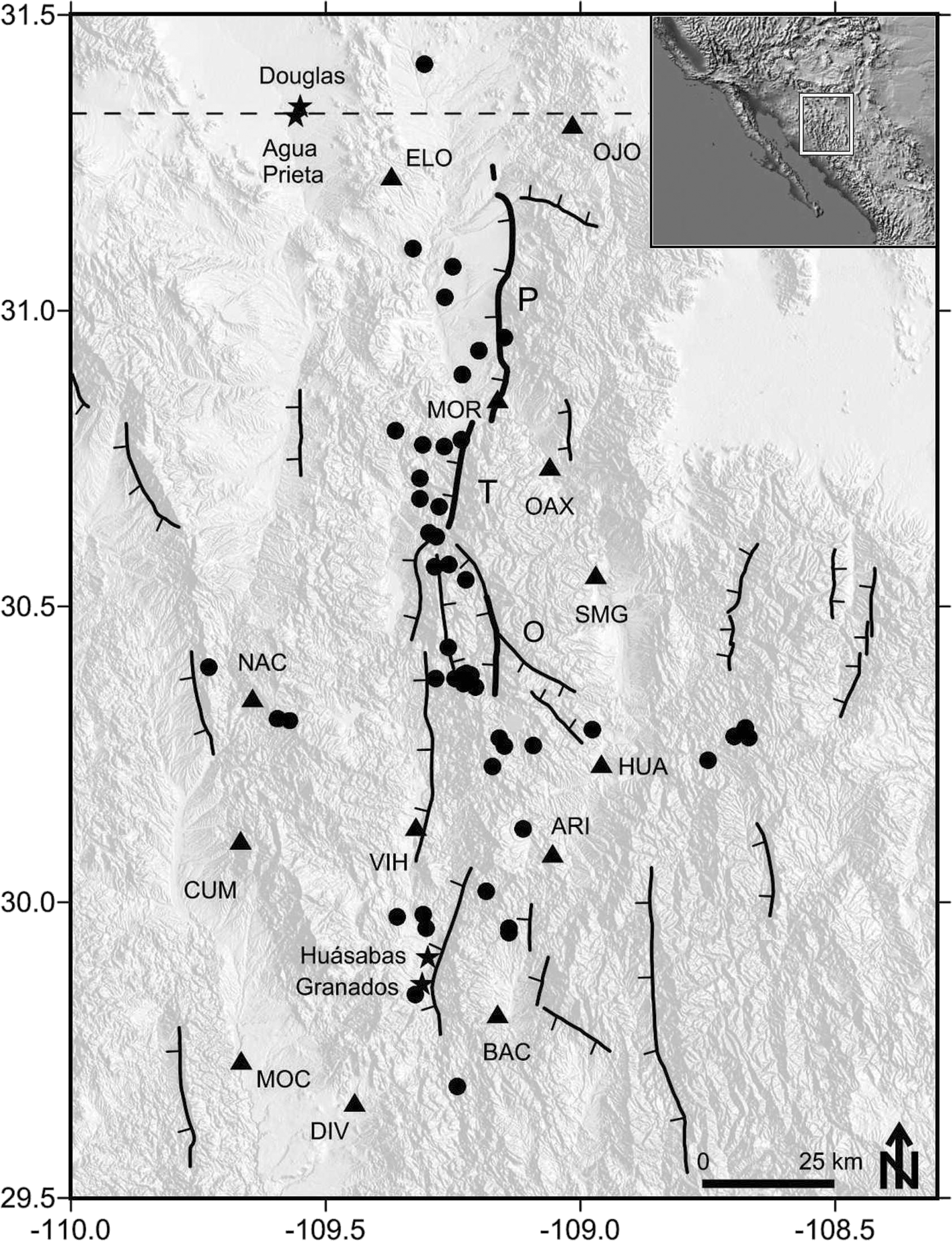

Supplement: Supplementary file 1 — Authors’ original file for figure 1 [file 40064_2014_1502_MOESM1_ESM.tif]

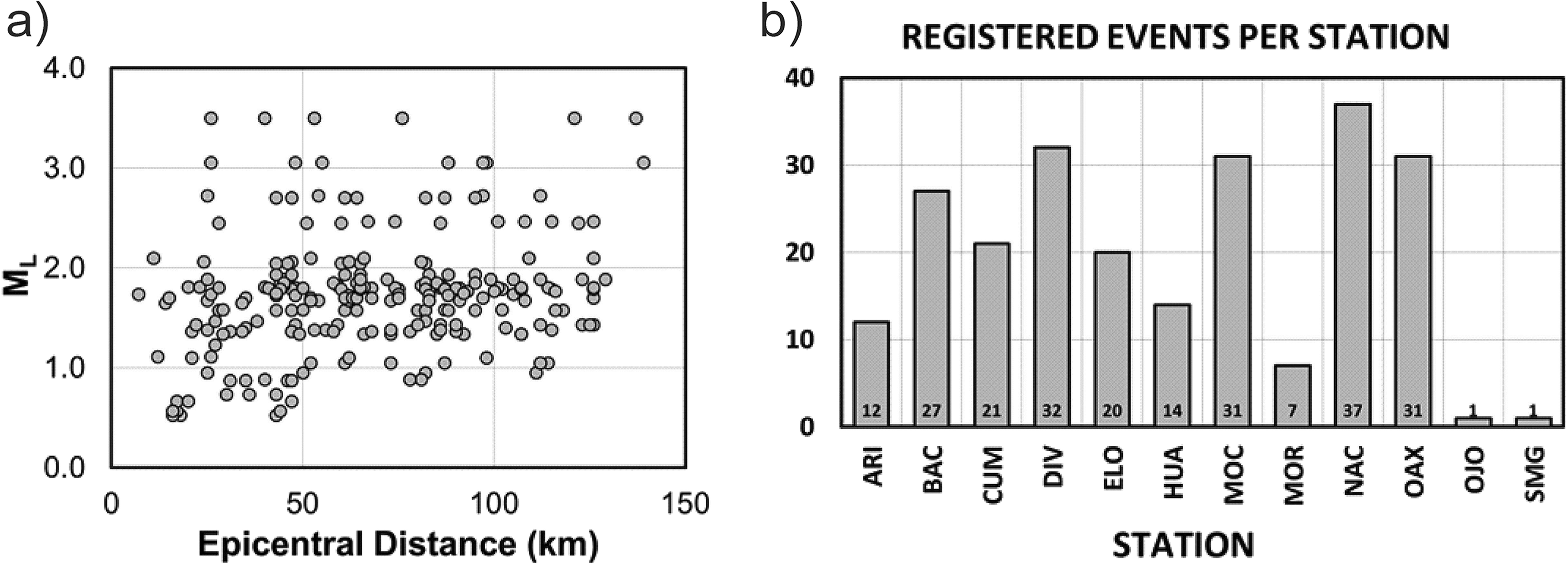

Supplement: Supplementary file 2 — Authors’ original file for figure 2 [file 40064_2014_1502_MOESM2_ESM.tif]

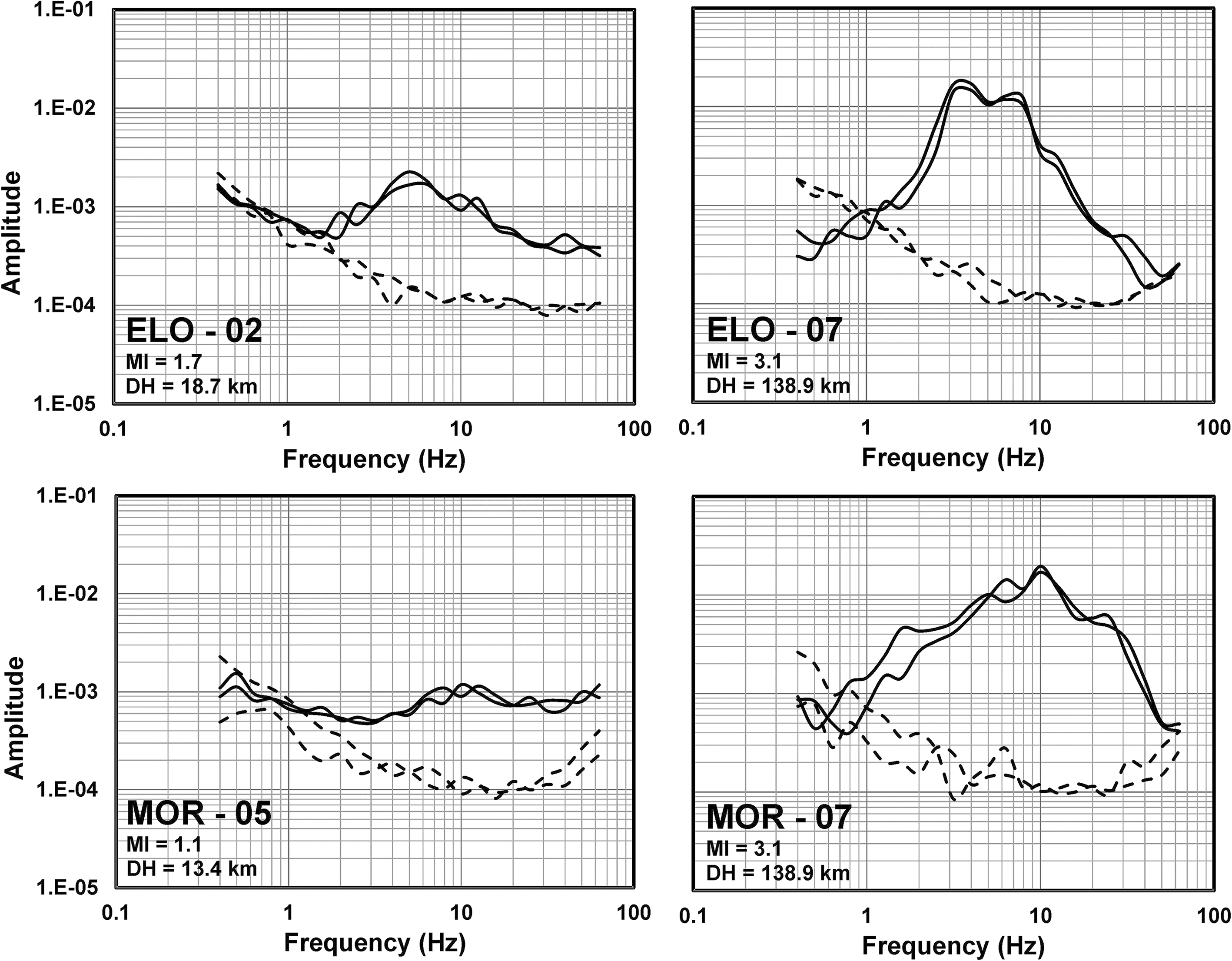

Supplement: Supplementary file 3 — Authors’ original file for figure 3 [file 40064_2014_1502_MOESM3_ESM.tif]

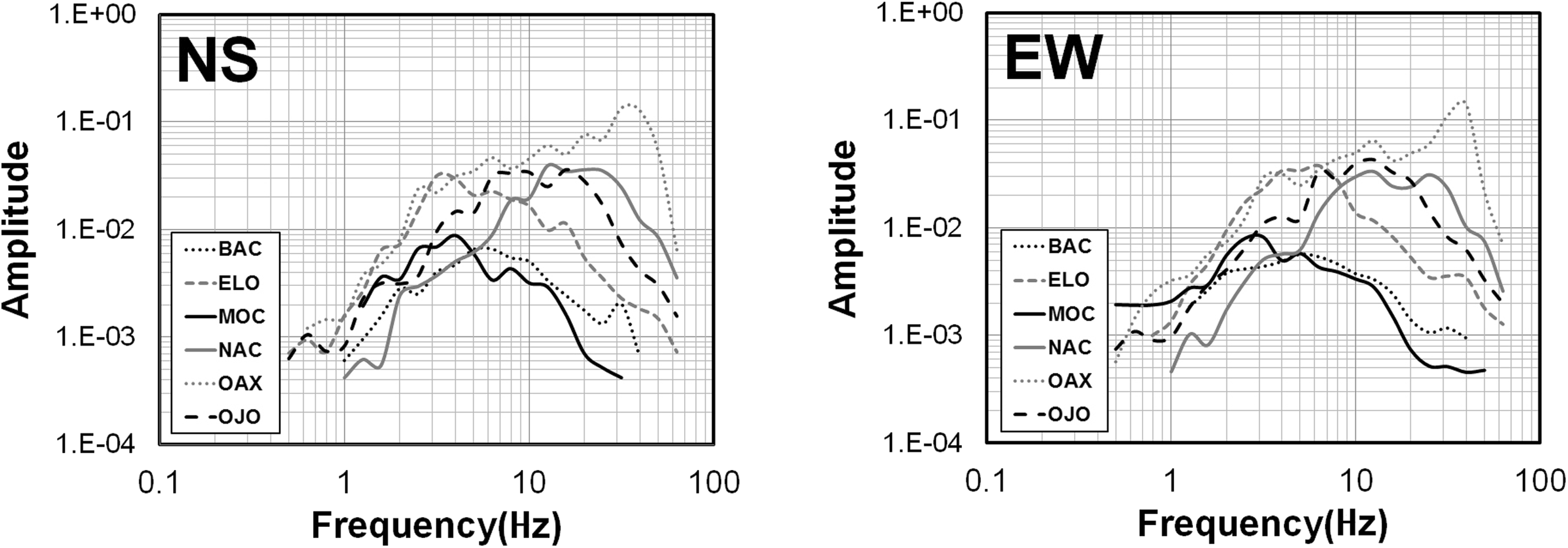

Supplement: Supplementary file 4 — Authors’ original file for figure 4 [file 40064_2014_1502_MOESM4_ESM.tiff]

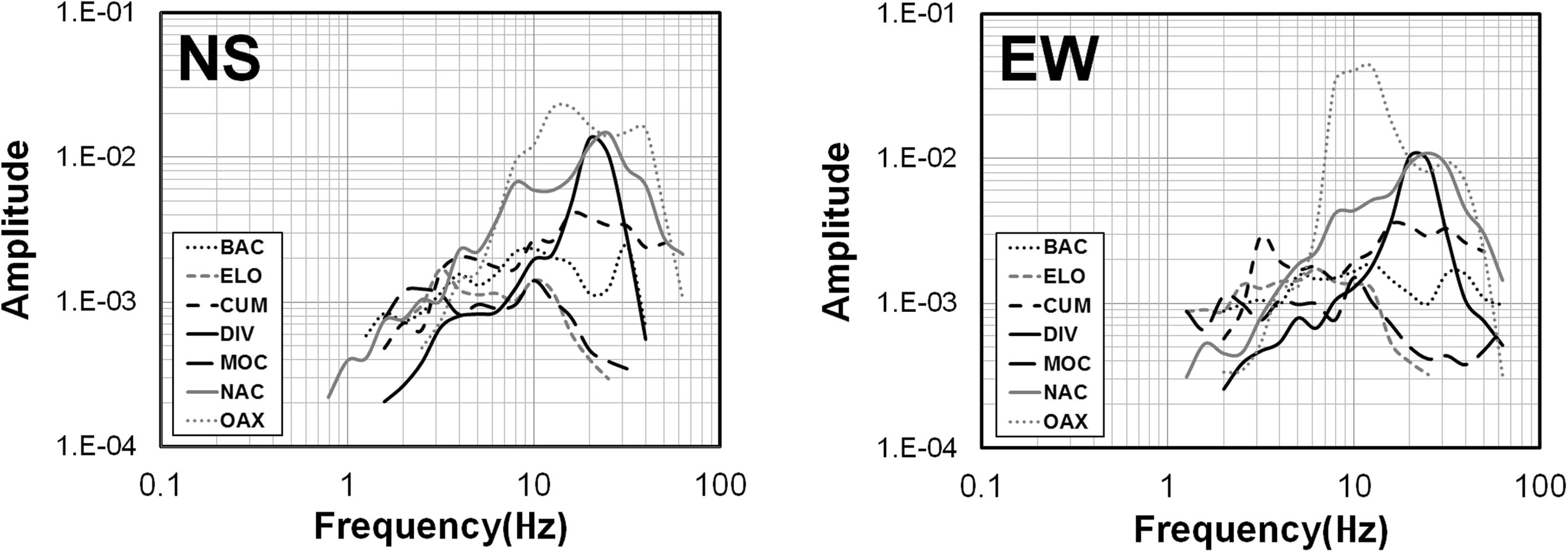

Supplement: Supplementary file 5 — Authors’ original file for figure 5 [file 40064_2014_1502_MOESM5_ESM.tiff]

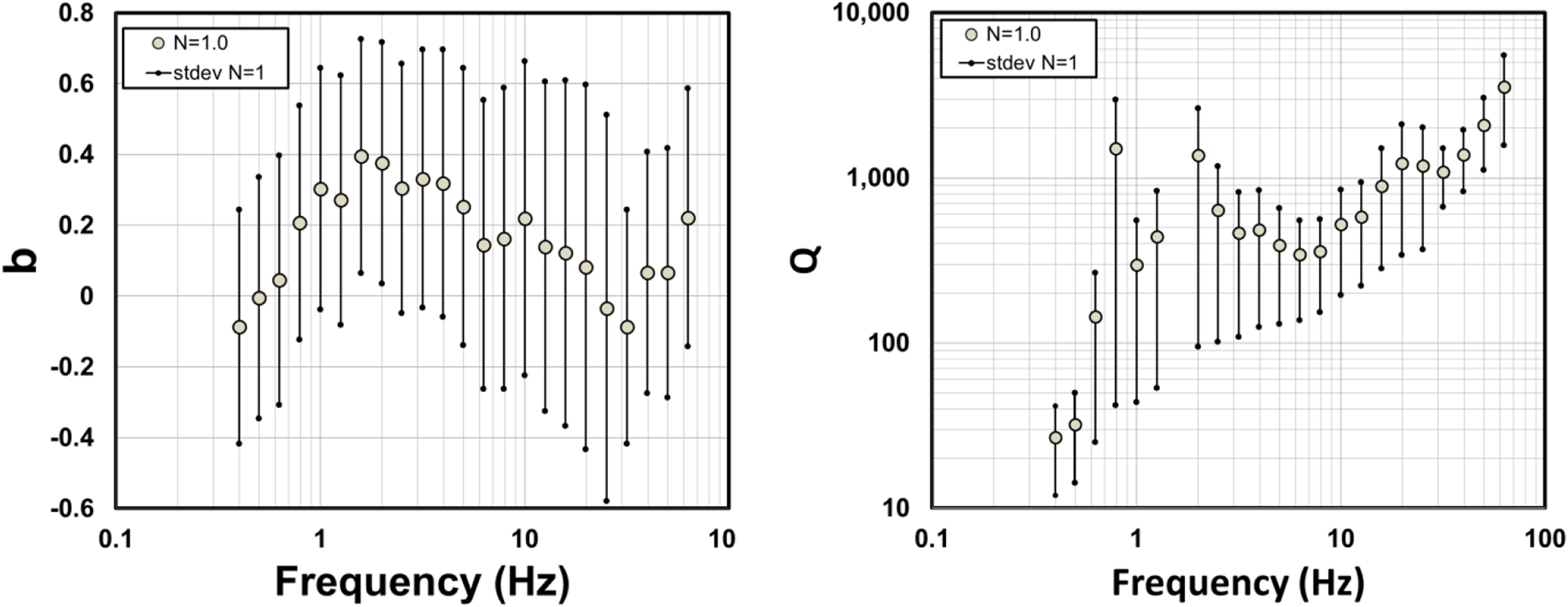

Supplement: Supplementary file 6 — Authors’ original file for figure 6 [file 40064_2014_1502_MOESM6_ESM.tif]

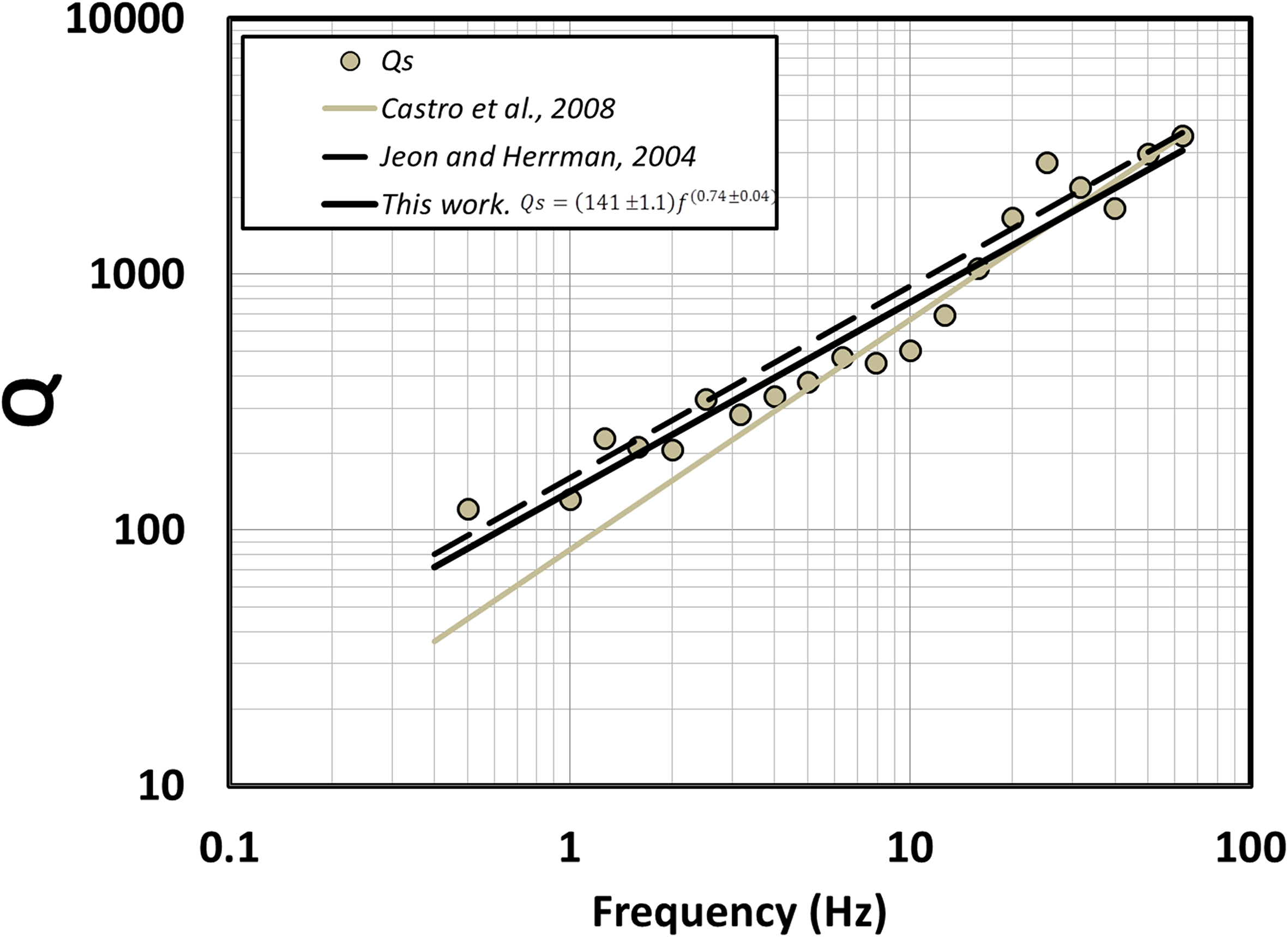

Supplement: Supplementary file 7 — Authors’ original file for figure 7 [file 40064_2014_1502_MOESM7_ESM.tif]

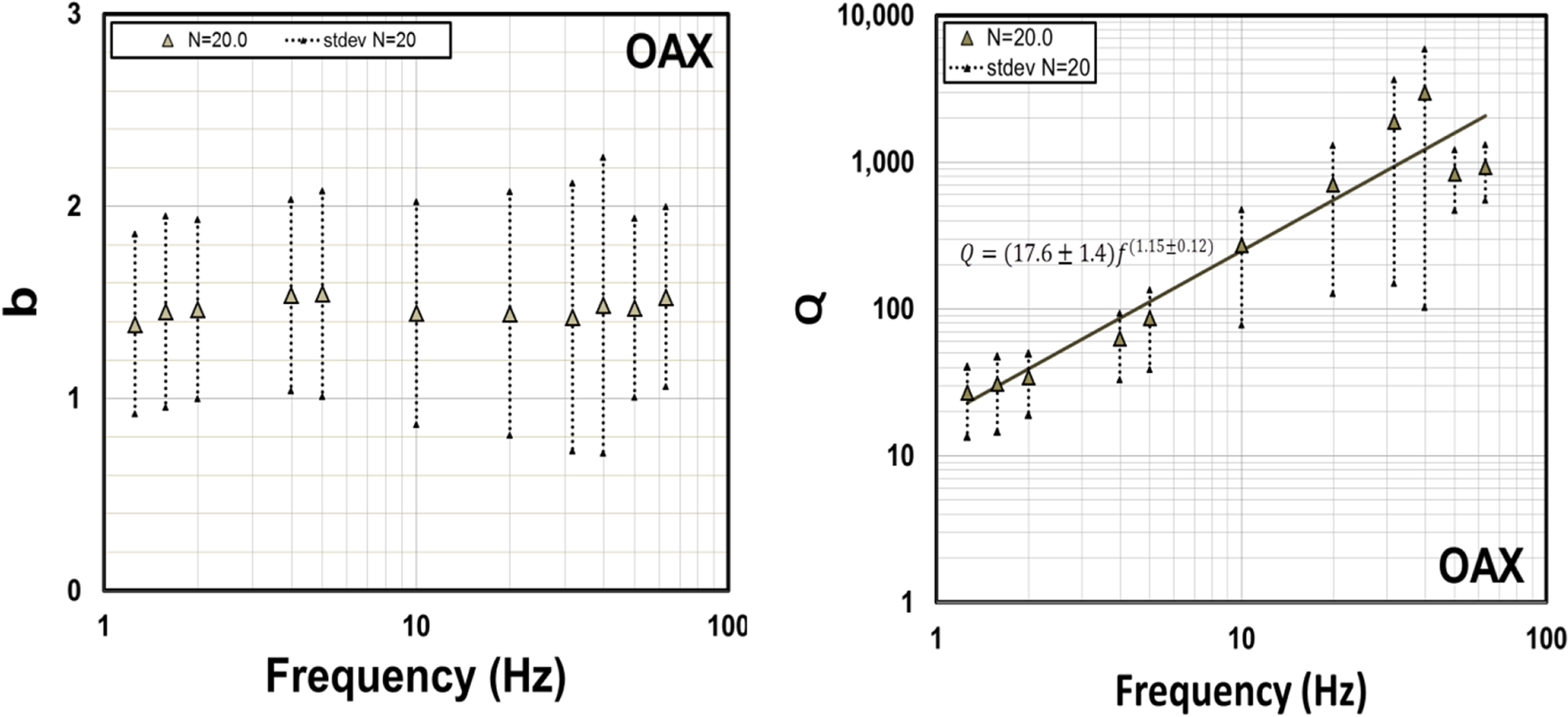

Supplement: Supplementary file 8 — Authors’ original file for figure 8 [file 40064_2014_1502_MOESM8_ESM.tif]

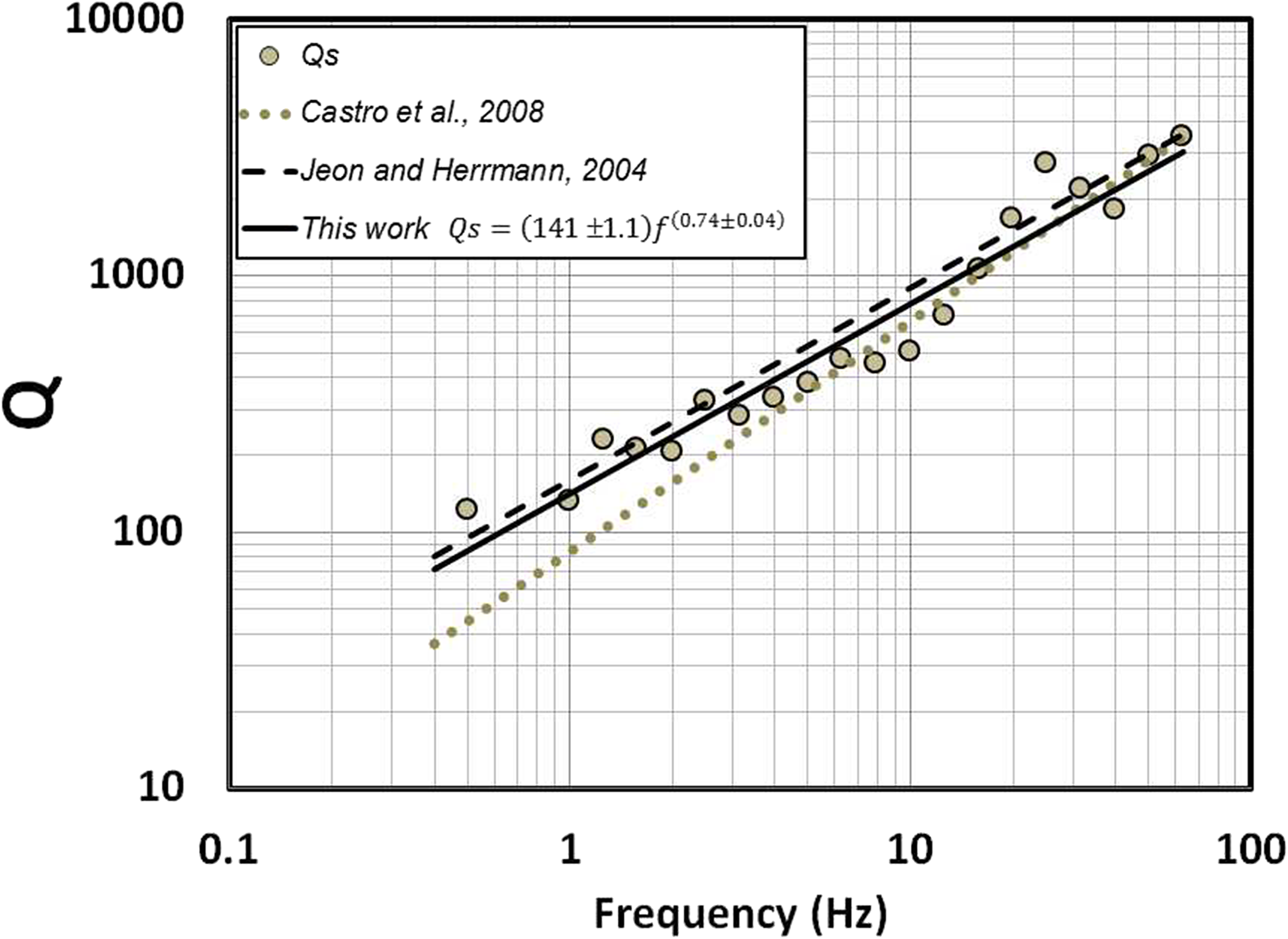

Supplement: Supplementary file 9 — Authors’ original file for figure 9 [file 40064_2014_1502_MOESM9_ESM.tif]

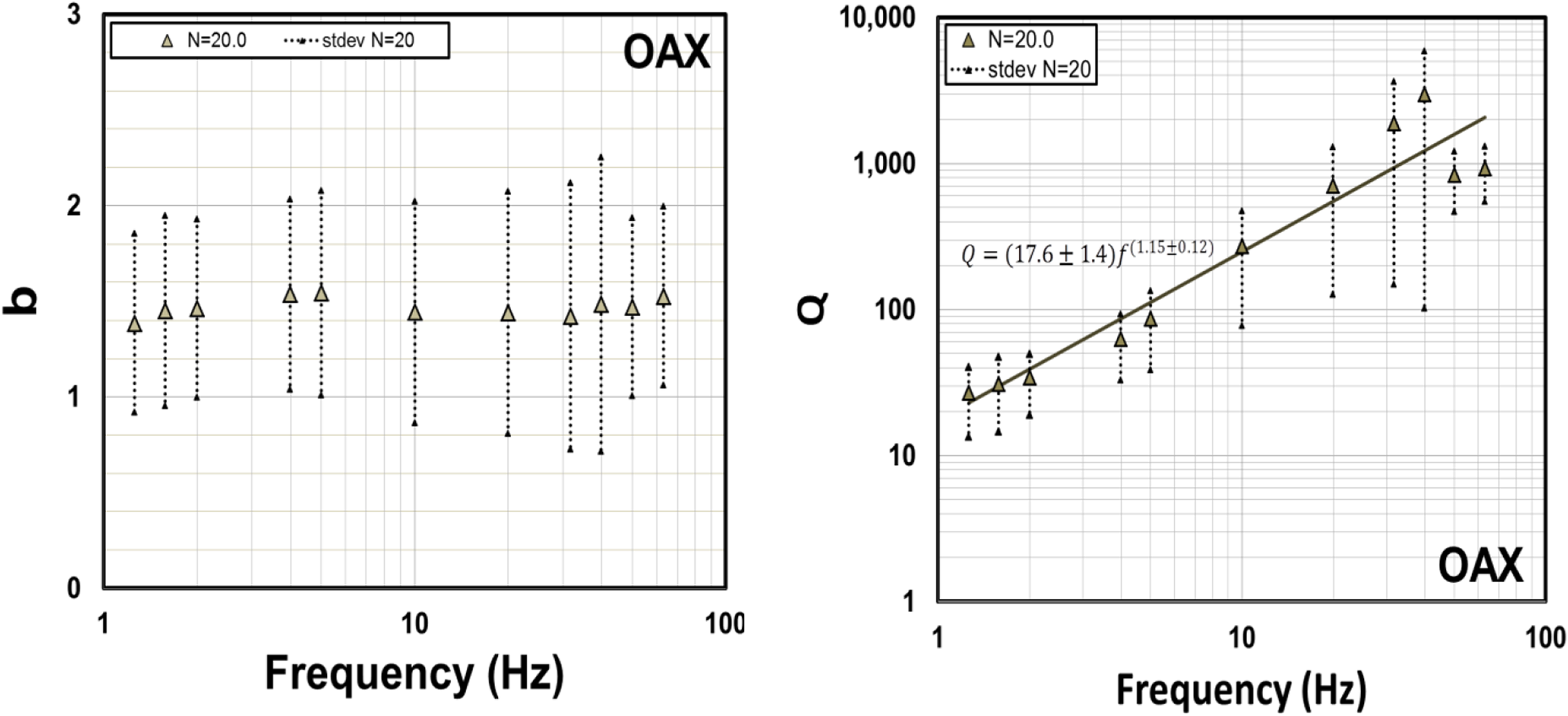

Supplement: Supplementary file 10 — Authors’ original file for figure 10 [file 40064_2014_1502_MOESM10_ESM.tif]

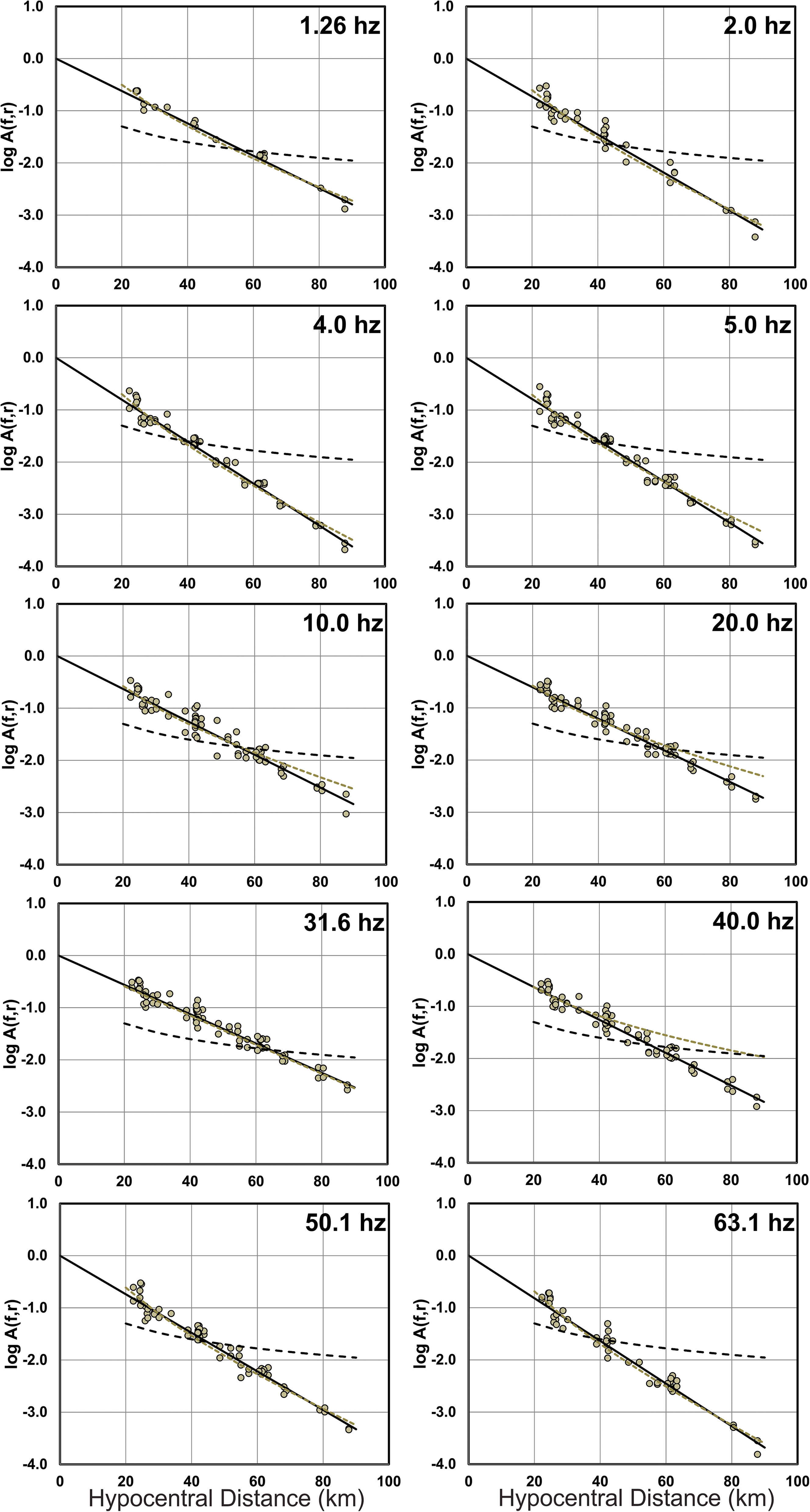

Supplement: Supplementary file 11 — Authors’ original file for figure 11 [file 40064_2014_1502_MOESM11_ESM.tif]
